# Myofibrillar Lattice Remodeling Is a Structural Cytoskeletal Predictor of Diaphragm Muscle Weakness in a Fibrotic *mdx* (*mdx Cmah^−/−^*) Model

**DOI:** 10.3390/ijms231810841

**Published:** 2022-09-16

**Authors:** Paul Ritter, Stefanie Nübler, Andreas Buttgereit, Lucas R. Smith, Alexander Mühlberg, Julian Bauer, Mena Michael, Lucas Kreiß, Michael Haug, Elisabeth Barton, Oliver Friedrich

**Affiliations:** 1 Institute of Medical Biotechnology, Department of Chemical and Biological Engineering, Friedrich-Alexander-University Erlangen-Nürnberg, Paul-Gordan-Str. 3, 91052 Erlangen, Germany; 2 Department of Neurobiology, Physiology and Behavior, University of California, Davis, CA 95618, USA; 3 College of Health & Human Performance, University of Florida, Gainesville, FL 32611, USA; 4 School of Medical Sciences, University of New South Wales, Wallace Wurth Building, 18 High Str., Sydney, NSW 2052, Australia

**Keywords:** muscular dystrophy, skeletal muscle, multiphoton microscopy, verniers density, cosine angle sum

## Abstract

Duchenne muscular dystrophy (DMD) is a degenerative genetic myopathy characterized by complete absence of dystrophin. Although the *mdx* mouse lacks dystrophin, its phenotype is milder compared to DMD patients. The incorporation of a null mutation in the *Cmah* gene led to a more DMD-like phenotype (i.e., more fibrosis). Although fibrosis is thought to be the major determinant of ‘structural weakness’, intracellular remodeling of myofibrillar geometry was shown to be a major cellular determinant thereof. To dissect the respective contribution to muscle weakness, we assessed biomechanics and extra- and intracellular architecture of whole muscle and single fibers from *extensor digitorum longus* (EDL) and diaphragm. Despite increased collagen contents in both muscles, passive stiffness in *mdx Cmah${}^{-/-}$* diaphragm was similar to *wt* mice (EDL muscles were twice as stiff). Isometric twitch and tetanic stresses were 50% reduced in *mdx Cmah${}^{-/-}$* diaphragm (15% in EDL). Myofibrillar architecture was severely compromised in *mdx Cmah${}^{-/-}$* single fibers of both muscle types, but more pronounced in diaphragm. Our results show that the *mdx Cmah${}^{-/-}$* genotype reproduces DMD-like fibrosis but is not associated with changes in passive visco-elastic muscle stiffness. Furthermore, detriments in active isometric force are compatible with the pronounced myofibrillar disarray of the dystrophic background.

## 1. Introduction

Duchenne Muscular Dystrophy (DMD) is caused by the absence of dystrophin and a disruption of the associated glycoprotein complex [1]. DMD patients suffer from heightened contraction-induced muscle injury which leads to de- and regeneration cycles [2,3]. The prolonged repetition of failed and improper muscle repair eventually promotes muscle inflammation, cell dysfunction, increased fiber branching, and impaired membrane stability [4]. While supporting therapies can ease and delay the disease progression, there is currently no ultimate cure [5,6,7]. Bulfield et al. (1984) described the *mdx* mouse model, which mimics the pathology of DMD in humans. This mouse model is characterized by a spontaneous nonsense mutation, leading to the loss of dystrophin, and shares many hallmarks with the human disease, including muscle weakness and fragility, ongoing degeneration and regeneration, and fibrotic remodeling of muscle, particularly in the diaphragm [8]. Although the *mdx* mouse historically served as the standard animal model to investigate causes and potential cures for DMD, the murine *mdx* phenotype is much milder than human or canine forms [9], particularly on the C57Bl10 background due to the upregulation of other cytoskeletal proteins, i.e., utrophin, to provide cellular stability [10], as well as a combination of genetic modifiers that alter disease progression. As a result of this, the development and validation of more severe mouse models of DMD have helped to provide more accurate disease patterns in the mouse which may accelerate the evaluation of therapies, including ablation of utrophin expression and using different background strains [11,12]. Of particular interest is a mouse model harboring an inactivating mutation in the *Cmah* gene, encoding for the CMP-Neu5Ac hydroxylase, which is an essential enzyme for the biosynthesis of N-glycolylneuraminic acid [13,14]. This mutation mimics DMD in humans, and the loss of activity results in greater fibrosis and poorer functional capacity, due to alterations in the sialic acid composition of glycoproteins surrounding the cells [15]. Consequently, diaphragms of 6-month-old *mdx Cmah${}^{-/-}$* mice present more fibrosis than diaphragms from age-matched *mdx* mice, accompanied by reduced strength in both diaphragms and limb muscles [13]. Both *mdx* and *mdx Cmah${}^{-/-}$* diaphragms are known to be highly dysfunctional and increasingly affected by fibrosis, necrosis and inflammation [13]. Limb muscles are likewise affected, but less than the diaphragm. Both dystrophic mouse models have already been extensively described on a macroscopic level [15], but a structure-function analysis with a single fiber and tissue 3D morphometric characterization has, to the best of our knowledge, not yet been performed. To address this, we applied SHG microscopy, which can reveal disease-related remodeling patterns in the sarcomeric microarchitecture of native, unstained tissue due to the non-centrosymmetric structure of the biopolymers myosin and collagen [16]. We further optimized a feature extraction algorithm, originally introduced by Friedrich et al. (2010) and Garbe et al. (2012), to quantitatively evaluate skeletal muscle fiber morphometry and to obtain a projected estimate for structure-related force production or impairment thereof in case of myofibrillar misalignment [17,18,19]. We quantified the structural severity of *mdx Cmah${}^{-/-}$* single muscle fibers by assessing (i) the cosine angle sum, which is defined as the overall orientation of sarcomeres within the longitudinal sarcomere lattice array of a myofiber, with respect to the main fiber axis, and (ii) verniers density, which describes the local axial lattice disruption between single myofibrils [18,19,20]. In the current study, altered cytoarchitecture of dystrophic *mdx Cmah${}^{-/-}$* muscle fibers was quantified by using label-free multiphoton microscopy and linked, for the first time, to passive and active force-generation in *mdx Cmah${}^{-/-}$* EDL and diaphragm muscle.

## 2. Results

**Figure 1:**
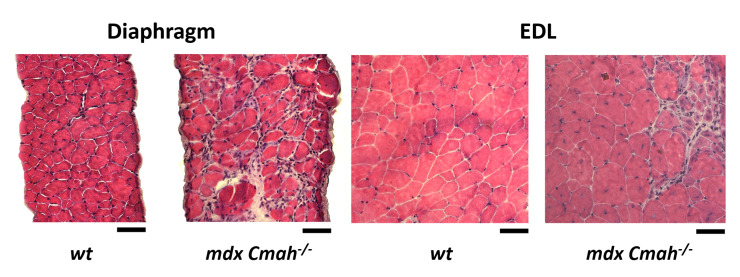
Extensive fibrosis in H&E muscle cross-sections of EDL and diaphragm muscle from *mdx Cmah${}^{-/-}$* mice. Increased fibrosis and scar tissue is observed in *mdx Cmah${}^{-/-}$* diaphragm, compared to *mdx Cmah${}^{-/-}$* EDL muscle (Scale bar 50 μm).

The *mdx Cmah${}^{-/-}$* genotype is known to more accurately reflect the human disease phenotype of DMD in comparison to the *mdx* phenotype. These differences manifest in similar fibrosis across muscle types and increased muscle weakness resulting in decreased individual life span [13]. In accordance with increased fibrosis in *mdx Cmah${}^{-/-}$* mice, initial H&E staining confirmed abundant collagen deposition in the diaphragm muscle, which is much more confined to singular regions in the EDL (Figure 1).

**Figure 2:**
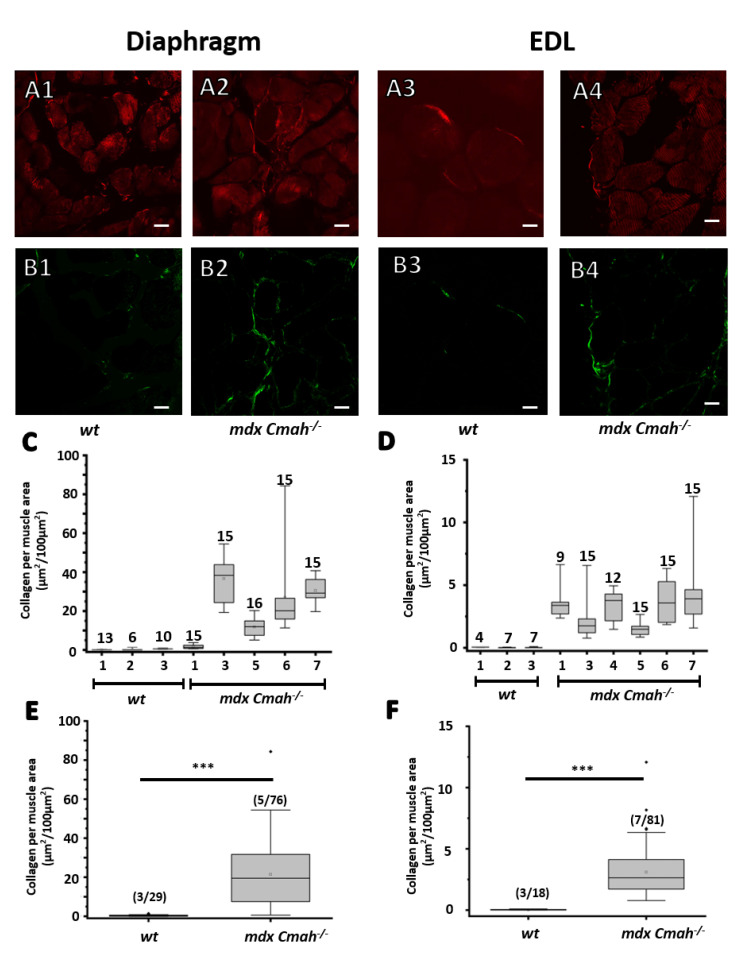
Marked fibrosis in *mdx Cmah${}^{-/-}$* diaphragm and EDL muscle sections assessed through label-free SHG morphometry. (**A**–**C**) Imaged and quantified collagen area per muscle tissue area. SHG signals are collected in forward scattered direction and collect both residuals of myosin-II and collagen (**A1**–**A4**). The myosin area was extracted via the measured autofluorescence in backward scattered direction and subtracted from the SHG signal resulting in quantifiable collagen areas (**B1**–**B4**). *mdx Cmah${}^{-/-}$* diaphragms tend to be highly fibrotic (**C**) more so than *mdx Cmah${}^{-/-}$* EDL (**D**). Combined results demonstrate a highly significant difference in collagen content per muscle area (**E**,**F**). Note the different ordinate scales in (**C**–**F**) (scale bar: 20 μm, *** *p* < 0.001, (m/n)) relate to animals/cryosections.

Furthermore, we applied label-free SHG multiphoton microscopy to quantify the collagen content in muscle cross-sections. The measured back-scattered autofluorescence signal was subtracted from the forward-scattered (Figure 2A) SHG signal, resulting in a well quantifiable collagen area (Figure 2B). The muscle area was extracted from the autofluorescence signal.

Especially *mdx Cmah${}^{-/-}$* diaphragm tissue revealed substantial fibrosis, whereas *mdx Cmah${}^{-/-}$* EDL sections only displayed minor to spotted collagen accumulations. Compared to their *wt* control, both *mdx Cmah${}^{-/-}$* EDL and *mdx Cmah${}^{-/-}$* diaphragm showed a significantly higher collagen content, and thus, fibrosis (EDL: 3.1% vs. 0.02%, *p* < 0.001, t = 6.9; Diaphragm 21.4% vs. 0.35%: *p* < 0.001, t = 6.814). Collagen accumulation is a known predominant reason for increased tensile strength [21,22]. However, results in the literature concerning the effect of collagen on active and passive force are highly controversial [21,23,24,25], highlighting the need for a more comprehensive investigation.

**Figure 3:**
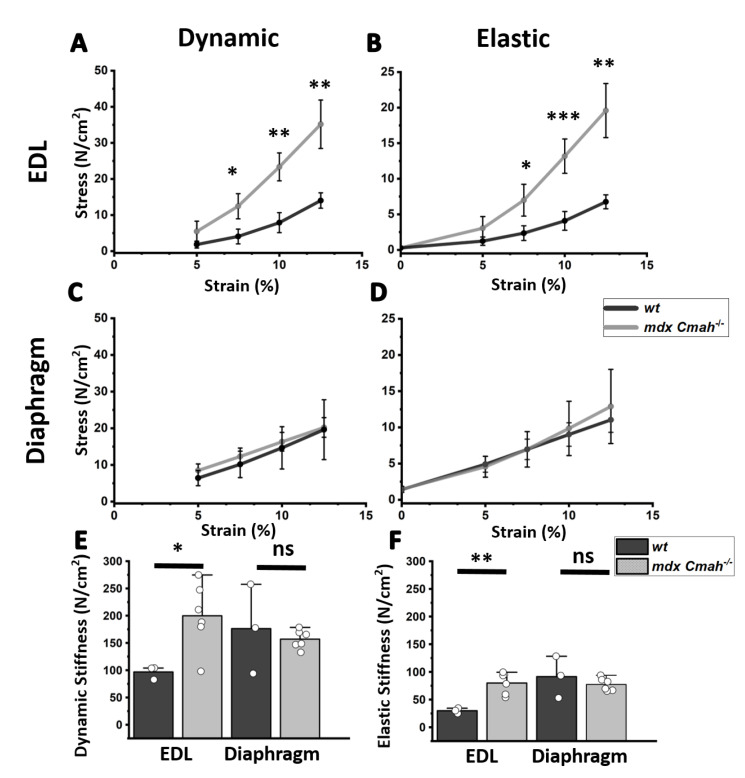
Passive visco-elastic stiffness is markedly increased in *mdx Cmah${}^{-/-}$* EDL but not in diaphragm muscles. Passive stresses (i.e., specific forces) of whole EDL (top panels, **A**,**B**) and diaphragm strips (central panels, **C**,**D**) from age-matched *wt* (n = 3) and *mdx Cmah${}^{-/-}$* mice (n = 6). EDL muscles from *mdx Cmah${}^{-/-}$* mice present with significantly higher stress values for the same strain. This results in increased dynamic and elastic stiffness in the EDL muscles but not the diaphragm muscles (**E**,**F**). Data are means ± SD. *, *p* < 0.1, **, *p* < 0.01, ***, *p* < 0.001 for comparisons between *wt* and *mdx Cmah${}^{-/-}$* muscles. ns, not significant.

**Figure 4:**
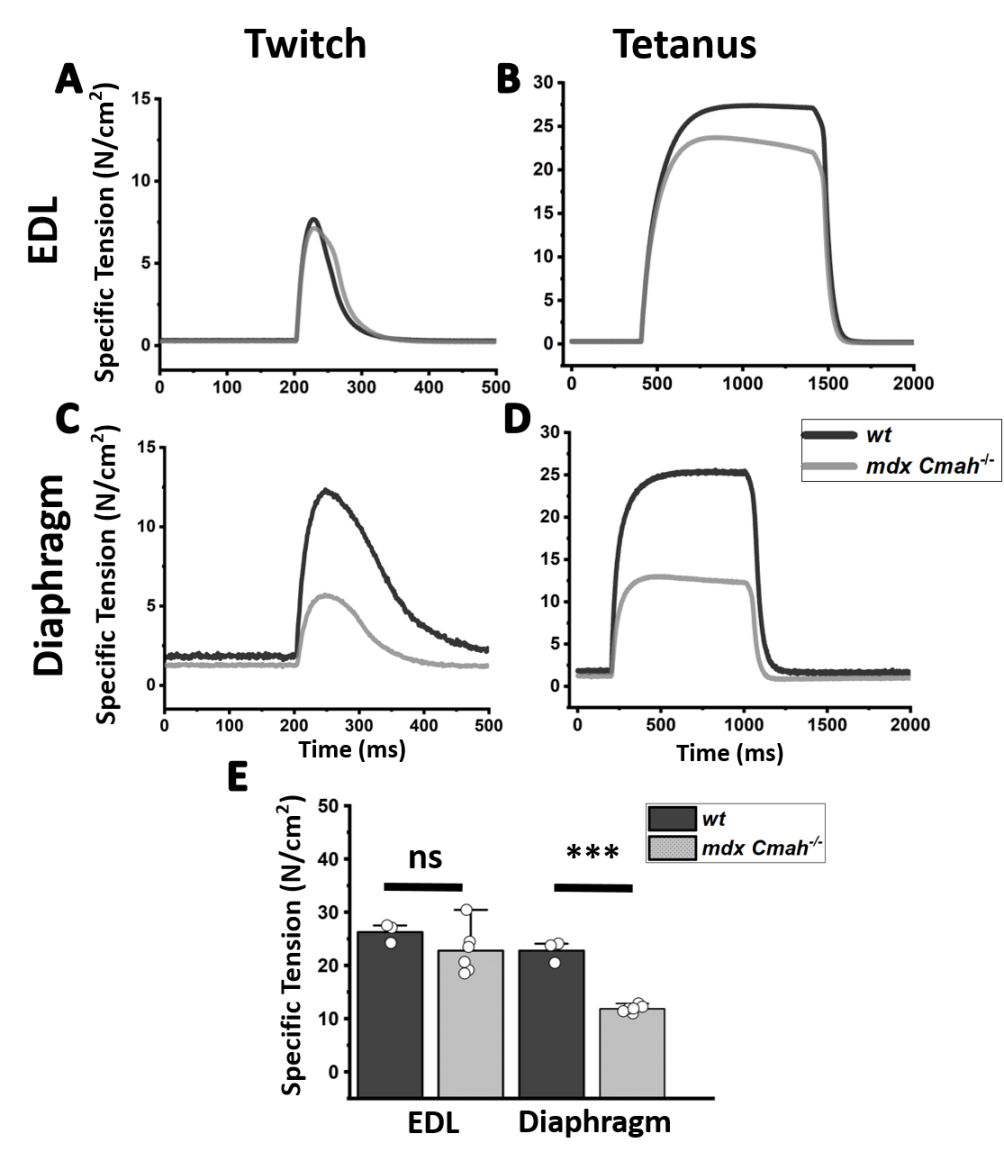
Specific active tension is markedly diminished in *mdx Cmah${}^{-/-}$* diaphragm but not in EDL muscles. Isometric contractile specific tension of EDL muscle (top panels, **A**,**B**) and diaphragm (central panels, **C**,**D**) from age-matched *wt* (n = 3) and *mdx Cmah${}^{-/-}$* mice (n = 6). Examples of specific tension traces are shown for a single twitch and for tetanic stimulation. Bottom panel (**E**) shows specific tension for tetanic stimulation (mean ± SD) (***: *p* < 0.001).

To elucidate the potential contribution of collagen accumulations to the fiber’s stress adaptation and passive biomechanics properties, a stress-relaxation protocol was carried out, as previously described [21]. Results suggest *mdx Cmah${}^{-/-}$* EDL muscle to be significantly stiffer when stretched beyond 105% L${}_{0}$ (Figure 3). Hereby, stress was separated into dynamic (initial exerted maximum force) and elastic (steady-state force after relaxation) stress to distinguish between initial restoration forces and visco-elastic force adaptation. These were calculated by applying the tangent of the quadratic fit at 10% stretch, as given by Smith et al. (2014) [21]. Dynamic and elastic (visco-elastic) stiffness doubled in *mdx Cmah${}^{-/-}$* EDL, whereas the *mdx Cmah${}^{-/-}$* diaphragm stiffnesses remained largely unaffected. Since it is known that fibrosis is more severe in *mdx Cmah${}^{-/-}$* diaphragm over limb muscle [13], we tested the hypothesis that twitch and tetanic isometric force in the genetic gain-of-function ablation for fibrosis also reproduced the overt difference in these two muscle types (i.e., recurrently active diaphragm over sporadically active EDL muscle). Diaphragm *mdx Cmah${}^{-/-}$* strips revealed a significant (*p* < 0.001) loss of tetanic and twitch peak-force over *wt* samples, with a specific tension that denotes only 50% of the control level (Figure 4). The observed relative loss of specific tension is similar to results reported for *mdx* diaphragm muscle [26]. Much in contrast to the force loss in diaphragm muscle, no muscle weakening was found in EDL.

**Figure 5:**
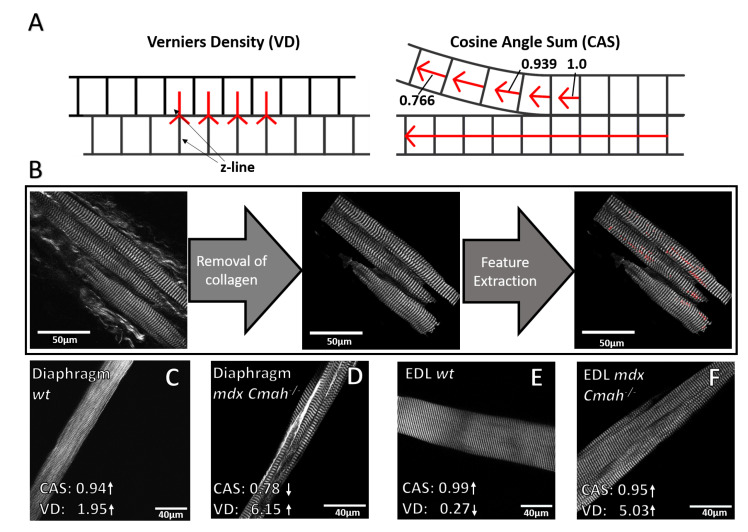
An improved algorithm allows to quantify structural remodeling in enhanced fibrosis-dystrophic *mdx Cmah${}^{-/-}$* EDL and diaphragm muscle. (**A**) CAS is defined by the integrated local sarcomere orientation with respect to the main fiber axis, pointing towards angular deviations from the perfectly parallel aligned pattern for values below 1. VD provides the number of lattice shifts between neighboring myofibrils (‘Y’ patterns) normalized to the imaged fiber area. (**B**) Due to extensive fibrosis in *mdx Cmah${}^{-/-}$* muscle, an improved algorithm allows further preprocessing steps to detect and extract Verniers and CAS. (**C**,**E**) High CAS and low VD as an indicator for ordered myofibrillar ultrastructure, as seen in a healthy myofiber taken from *wt* diaphragm and EDL, respectively. (**D**) Example of a highly fibrotic and remodeled *mdx Cmah${}^{-/-}$* diaphragm single muscle fiber showing numerous myofibrillar lattice shifts and a high variation in sarcomere orientation, leading to a low CAS and a high VD (as indicated). A substantial amount of extracellular collagen streaks can be seen around the fiber. (**F**) Example of an *mdx Cmah${}^{-/-}$* EDL myofiber with orderly orientation despite a marked myofibrillar lattice shift, reflected by a high CAS and a high VD value. Units: CAS (-), VD ($\frac{\#}{100\mu m^{2}}$).

Although fibrosis is commonly seen as a hallmark in chronic muscle tissue remodeling in muscular dystrophy [27,28], a much less known remodeling pattern affecting the evenly spaced myofibrillar microarchitecture (myofibrillar lattice array) has been documented in *mdx* muscle in recent years [18,19,20]. These studies mostly focused on limb muscle fibers, either in single fiber studies [17] or assessment of fibers within thick tissue sections [29]. The extent of contractile apparatus remodeling in diaphragm is less well documented, if at all. Therefore, we measured changes in serial sarcomere lattice orientation (cosine angle sum, CAS) and sarcomere shifts between adjacent single myofibrils (verniers density, VD) in *mdx Cmah${}^{-/-}$* mice. We hypothesized that despite increased overall fibrosis, myofibrillar remodeling was again more severe in the mechanically more active diaphragm over the sporadically used EDL muscle. This was assessed by recording label-free image stacks of single muscle fibers and extracting the CAS and VD based on the 3D entity of image stacks. Figure 5A visualizes the effects of CAS and VD on skeletal muscle single fibers. As for the results above, collagen signals were again removed in a pre-processing step (Figure 5B). As shown in Figure 5C,E, the CAS of *wt* muscle is characterized by values close to 1 with low VDs. This indicates parallelly aligned myofibrils that are in-register. Dystrophic remodeling as in *mdx Cmah${}^{-/-}$* fibers leads to a reduced CAS but increased VD values, demonstrating a loss of ordered angular myofibrillar orientation and an overall disturbance in myofiber registration.

**Figure 6:**
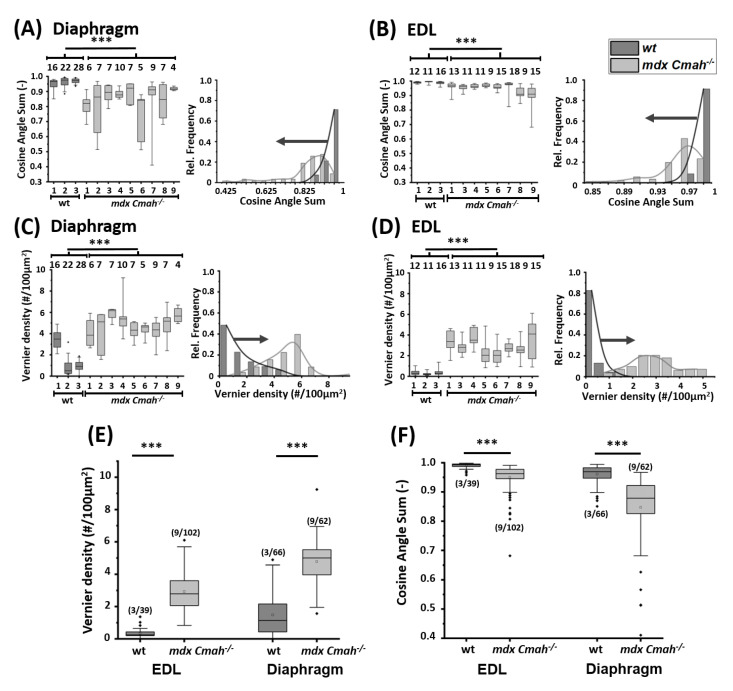
Myofibrillar cytoarchitecture is severely compromised and disordered in single fibers from EDL and diaphragm from adult *mdx Cmah${}^{-/-}$* over *wt* mice. Diaphragm and EDL muscle fibers show a highly significant shift toward decreased CAS (**A**,**B**) and increased VD values in *mdx Cmah${}^{-/-}$* mice (**C**,**D**). Combined results are shown in (**E**,**F**). Abscissa numbers correspond to the individual label of each experiment (***: *p* < 0.001). (m/n) relates to animals/muscle single fibers.

Data from *mdx Cmah${}^{-/-}$* mice varied greatly between animals as well as across samples from the same animal, illustrating different levels of muscle remodeling from sample to sample. The distribution of morphometric parameters from *mdx Cmah${}^{-/-}$* EDL and diaphragm muscle fibers shifts to lower CAS (Figure 6A,B) and higher VD (Figure 6C,D). Overall, diaphragm single muscle fibers were more fibrotic and morphologically altered compared to EDL single fibers. Taking the entire population of EDL and diaphragm muscle fibers into account (Figure 6E,F), the collective results reinforce the observed effect of severely compromised cytoarchitecture in both muscles. This is systematically expressed in a significantly decreased CAS and increased VD in the *mdx Cmah${}^{-/-}$* phenotype compared to their *wt* counterpart for both EDL and diaphragm single muscle fibers, suggesting a markedly disordered cytoarchitecture and sarcomere lattice array; while a severe misalignment of sarcomeres was detectable for all *mdx Cmah${}^{-/-}$* muscle fibers, this effect was less pronounced in the sporadically active EDL, as opposed to the strong differences seen in permanently active diaphragm fibers.

## 3. Discussion

The molecular cytoarchitecture of muscles in DMD patients is known to change in morphology, and thus, function through repeated cycles of degeneration/regeneration and/or inflammation [2], leading to progressive muscle weakness and impaired functionality [22]. While both the traditional *mdx* and the more ‘humanized’ *mdx Cmah${}^{-/-}$* mouse model were used to resemble and investigate DMD, they do show pathologies of varied magnitude across different muscle types [13,30]. Due to the degenerative processes that are induced by a lack of dystrophin, the individual fascicle cross-sections tend to be mis-shaped [3]. These pathological changes have been widely discussed in the literature in limb muscles and present with the loss of their elliptical shape; thus, fibers are shifting to a rather randomly shaped appearance [31]. Much in agreement with those findings, we found the loss of structure in both H&E staining as well as in our SHG image analysis of diaphragm and EDL muscle. Particularly for EDL, the elliptical cross-section was altered towards rather ‘polygonal’ ones. Collagen quantification revealed increased content in *mdx Cmah${}^{-/-}$* diaphragm and EDL compared to their *wt* littermates. Especially the *mdx Cmah${}^{-/-}$* diaphragm muscle presented with up to ten times more collagen content compared to EDL. The increased fibrosis seen in diaphragm muscle may be explained through its periodic use-dependency during respiration. This is also in agreement with marked fibrosis in *mdx* over *wt* mice from picrosirius red staining, although the absolute collagen contents reported were much higher compared to our SHG analyses [21]. This can be explained by the effect of SHG responsiveness, which requires a certain spatial distribution and size of collagen fibrils, and as such, tiny or non-filament-like collagen deposits may not provide SHG signals [32]. Eventually, however, it remains controversial if increased fibrosis, such as seen in the *mdx* and *mdx Cmah${}^{-/-}$* mouse model, effectively increases the muscle fiber’s axial stiffness after all. In that regard, we find no significant alterations in neither dynamic nor elastic stiffness in *mdx Cmah${}^{-/-}$* compared to *wt* diaphragm single muscle fibers, which is in accordance with Smith et al. (2014), who did not find a significant correlation between increased collagen content and passive muscle stiffness in *mdx* mice. This suggests that fibrosis may not be the major factor for an increased fiber stiffness in dystrophic skeletal muscle [21]. In contrast to the passive biomechanics findings, we find a reduction in isometric peak force and specific tension in *mdx Cmah${}^{-/-}$* diaphragm, which can be attributed to a combination of high fibrosis (muscle fibers are replaced by scar tissue) and the observed cellular remodeling, leading to a misorientation of force vectors (reduced CAS) and unsynchronized sarcomere activation (high VD shifts). Intense cellular remodeling of the diaphragm may also be attributed to the reduced functionality of the dystrophin-associated glycoprotein (DAG) complex in *mdx Cmah${}^{-/-}$*, leading to a worsened extracellular matrix (ECM) binding [13]. In comparison, *mdx Cmah${}^{-/-}$* EDL fibers appear to be much stiffer than their *wt* counterparts, which is in accordance with reports of *mdx* muscles in the literature [21,33]. We, however, observed no loss of active force in both twitch and tetanic stimulation experiments of whole muscles. Dystrophic *mdx Cmah${}^{-/-}$* EDL muscle showed a significant cytoarchitecture remodeling, but less so compared to *mdx Cmah${}^{-/-}$* diaphragm. Additionally, results from *wt* EDL fibers were similar to previously described values in the literature, confirming the overall robustness of our employed imaging technique and analysis algorithm [34].

## 4. Materials and Methods

### 4.1. Animal Handling

Animal studies were performed in accordance with the Institutional Animal Care and Use Committee and approved by the University of Florida. The dystrophic *mdx* mouse harboring an inactivating mutation of the CMAH gene, encoding CMP-Neu5Ac hydroxylase, and strain-matched C57BL10 wild type controls were used [13]. All mice were males, 9 months of age, and were maintained in the animal facility in a 12-h light–dark cycle with access to food and water.

### 4.2. Muscle Preparation and Multiphoton Imaging

An inverted multiphoton system (TriM-Scope II; LaVision BioTec GmbH; Bielefeld, Germany) with a femtosecond pulsed Ti:Sa laser was used to image muscle samples (water immersion objective, LD C-Apochromat lens 40x/1.1/UV-VIS-IR/WD 0.62, Carl Zeiss, Jena, Germany) for myosin-II and collagen structures. The Ti:Sa laser was tuned to a wavelength of 810 nm. The signal in forward-scattered direction was collected through a band-pass filter at 405 nm ±10 nm (Chroma, Rockingham, VT, USA). The backscattered signal was collected for collagen area measurements. It was separated with a 460 nm beamsplitter, and both beams were collected. The autofluorescence (>460 nm) signal was used to separate myosin and collagen via thresholding from the forward scattered signal. The <460 nm channel images were collected for binary mask denoising, where applicable. Images were acquired as stacked images with a resolution of 1072 × 1072 pixels. Images were processed in Image J (National Institute of Health, Bethesda, MD, USA) to apply gamma correction and adjusted for brightness for enhanced visualization only.

### 4.3. Muscle Mechanics

Muscles were dissected from anesthetized mice for active and passive force measurements as previously described. [21] Briefly, EDL muscles and strips of diaphragm muscle were placed in oxygenated Ringer’s solution (119 mM NaCl, 4.74 mM KCl, 3.36 mM CaCl${}_{2}$, 1.18 mM KH${}_{2}$PO ${}_{4}$, 1.18 mM MgSO${}_{4}$, 25 mM HEPES, and 2.75 mM glucose) gas-equilibrated with 95% O${}_{2}$ and 5% CO${}_{2}$. After determining optimum length by a series of twitches, maximum tetanic force was measured and used to calculate specific force (force per physiological cross sectional area). Physiological cross sectional area was determined by the following equation:

(1) $$PCSA=\frac{m}{L_{0}\cdot \frac{L_{f}}{L_{0}}\cdot \rho }$$

where *m* is muscle mass (m), $L_{0}$ is muscle length, $\frac{L_{f}}{L_{0}}$ is the ratio of fiber length to optimal muscle length, and the density of muscle is ρ = 1.06 $\frac{g}{cm^{3}}$ [35]. $\frac{L_{f}}{L_{o}}$ was 0.45 for EDL and 1.0 for diaphragm. Next, dynamic and elastic passive forces were measured at 5–12.5% strain. Following mechanical measurements, muscles were blotted and weighed, fixed in 4% PFA, and then stored in PBS pH 7.4 for subsequent sectioning and imaging.

### 4.4. Single Muscle Fiber Preparation and Cryosections

Whole muscle tissue was fixed in 4% paraformaldehyde and transferred into PBS pH 7.4 (Gibco ThermoFisher, Waltham, Massachusetts, USA) on dry ice between laboratories. Single muscle fibers were manually dissected and separated using fine forceps and mounted in a custom-made measurement chamber with petroleum jelly. The remaining muscle tissue was cut laterally, and cryosections with a thickness of 10 μm were collected at the highest cross-sectional area. Each slice was investigated by multiphoton microscopy to determine collagen content.

### 4.5. Feature Extraction

A custom-made algorithm was used as described in Garbe et al. (2012) with modifications described and used as in Buttgereit et al. (2013) [19,20]. The analysis was adjusted for *mdx Cmah${}^{-/-}$* diaphragm samples to account for their substantial fibrosis. The script was used to extract VD and CAS. The algorithm was originally developed for the analysis of single fibers. In muscle tissues, especially those with high levels of fibrosis, SHG signals from the extracellular collagen are also recorded. This collagen SHG signal may interfere with CAS and VD. Therefore, it was necessary to separate the SHG signals of myosin from collagen with an additional tool. This separation tool uses the autofluorescence signal of the muscle cell as cellular reference to isolate the SHG signals of the myosin for the analysis.

## 5. Conclusions

Here, we compared fibrosis patterns and cytoarchitectural remodeling of the myofibrillar contractile apparatus and their effects on active and passive biomechanical performance in the *mdx Cmah${}^{-/-}$* mouse model that recreates a higher fibrotic phenotype than the original *mdx* model, similar to what is seen in DMD patients. Confirming previous studies on slow-twitch *mdx* muscles, diaphragm showed a more than ten times higher fibrosis than fast-twitch EDL muscle. However, this was not associated with an increased diaphragm stiffness, neither elastic nor viscous. On the other hand, the markedly compromised active force capacities (twitch and tetanus) in the diaphragm were compatible with the enhanced fibrosis, as well as with the much more pronounced alteration in myofibrillar geometry, as determined by SHG morphometry, supporting a mostly contractile apparatus structure-related ‘weakness’ that is more pronounced in *mdx Cmah${}^{-/-}$* diaphragms but only marginal in EDL muscles. We, therefore, conclude that intracellular contractile cytoarchitectural remodeling shall be seen as a reliable and complementary parameter to judge function from structure in cytopathological muscle studies rather than focusing on fibrosis alone.

## Data Availability

The data supporting the findings of this study are available from the corresponding author upon reasonable request.

## Abbreviations

The following abbreviations are used in this manuscript:

DMD	Duchenne Muscular Dystrophy
EDL	Extensor digitorum longus
SHG	Second harmonic generation
CAS	Cosine angle sum
VD	Verniers density
